# Adenomatoid Odontogenic Tumor Arising From a Dentigerous Cyst in a 10-Year-Old Patient: A Case Report and Literature Review

**DOI:** 10.7759/cureus.89583

**Published:** 2025-08-07

**Authors:** Syamprasad Padappayil, Arjun Krishnadas, Mahija Janardhanan, Ambuj Aggarwal, Pramod Subash

**Affiliations:** 1 Oral and Maxillofacial Surgery, School of Dentistry, Amrita Vishwa Vidyapeetham, Kochi, IND; 2 Craniomaxillofacial Surgery, Amrita Institute of Medical Sciences, Kochi, IND; 3 Oral Pathology and Microbiology, School of Dentistry, Amrita Vishwa Vidyapeetham, Kochi, IND

**Keywords:** adenomatoid odontogenic tumor, cyst enucleation, dentigerous cyst, marsupialization, maxillofacial surgery, oral and maxillofacial pathology

## Abstract

Adenomatoid odontogenic tumor (AOT) is a benign, well-encapsulated odontogenic lesion that typically presents as a slow-growing, asymptomatic mass. Surgical enucleation or curettage remains the treatment of choice due to the tumor’s non-invasive nature and well-defined borders, which facilitate complete removal with minimal risk of recurrence. Interestingly, some studies have suggested that AOTs may occasionally arise within pre-existing dentigerous cysts, indicating a possible developmental relationship between the two entities.

We report a case of a 10-year-old female patient presenting with a one-week history of swelling on the left side of the face. Clinical examination revealed a firm, tender swelling in the left upper buccal vestibule, extending from the maxillary lateral incisor to the second deciduous molar. The left maxillary canine was unerupted. Aspiration yielded straw-colored fluid, suggestive of a cystic lesion. Cone beam computed tomography (CBCT) showed a well-defined, unilocular radiolucency surrounding the crown of the unerupted canine, measuring approximately 2 × 2 cm and extending from the central incisor to the first molar. A provisional diagnosis of a dentigerous cyst was made. Surgical enucleation and removal of the impacted canine were performed under general anesthesia. Histopathological analysis confirmed an AOT arising from a dentigerous cyst.

## Introduction

Dentigerous cysts and adenomatoid odontogenic tumors (AOTs) are two distinct odontogenic lesions that share a complex relationship. Dentigerous cysts, the second most common type of odontogenic cyst, form around impacted teeth, particularly wisdom teeth, and can potentially transform into more complex lesions like ameloblastoma. AOTs, on the other hand, are rare, benign tumors often associated with impacted teeth [1]. 

In the 2005 WHO classification of odontogenic tumors, AOT was classified into the first group of tumors (odontogenic epithelium without ectomesenchyme). Because of the absence of ectomesenchyme in immunohistochemical staining, calcification and dysplastic dentin, AOT is now considered the result of a metaplastic process rather than epithelial-ectomesenchyme interaction [2].

The origin and nature of AOT are still under debate because it could be hamartomatous or a true benign neoplasm. AOT is classified into three distinct types: follicular, extrafollicular, and peripheral [3]. Follicular and extrafollicular variants are central (intraosseous) lesions, with the follicular type frequently misdiagnosed as a dentigerous cyst due to its similar radiographic presentation. Unlike more aggressive odontogenic lesions, AOT typically causes displacement of adjacent teeth rather than root resorption.

The follicular variant often mimics a dentigerous cyst, as it is associated with the crown of an unerupted tooth. The extrafollicular type may resemble a residual cyst radiographically. In contrast, the peripheral variant, which occurs in the gingival soft tissues, can clinically mimic a gingival fibroma [2].

## Case presentation

**Figure 1:**
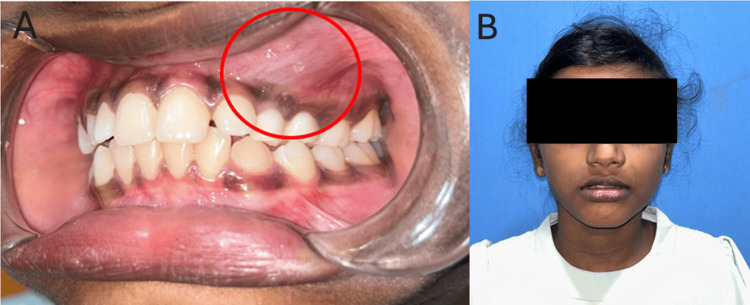
Preoperative images (A) A firm, tender swelling was noted over the left upper buccal vestibule, extending from the left upper lateral incisor to the second deciduous molar (highlighted in red circle). (B) Obliteration of the nasolabial fold was noted on the left side.

A 10-year-old female patient presented with a chief complaint of swelling on the left side of her face, which had been progressively increasing over the past week. Additionally, there was a noted delay in the eruption of the left maxillary canine. Initial assessment at a local hospital included radiographic imaging - cone beam computed tomography (CBCT) and an orthopantomogram (OPG). On clinical examination, a firm and tender swelling was observed in the left upper buccal vestibule. The swelling extended anteroposteriorly from the region of the left maxillary lateral incisor to the second deciduous molar, suggestive of an underlying pathological process associated with the unerupted canine (Figure 1).

**Figure 2:**
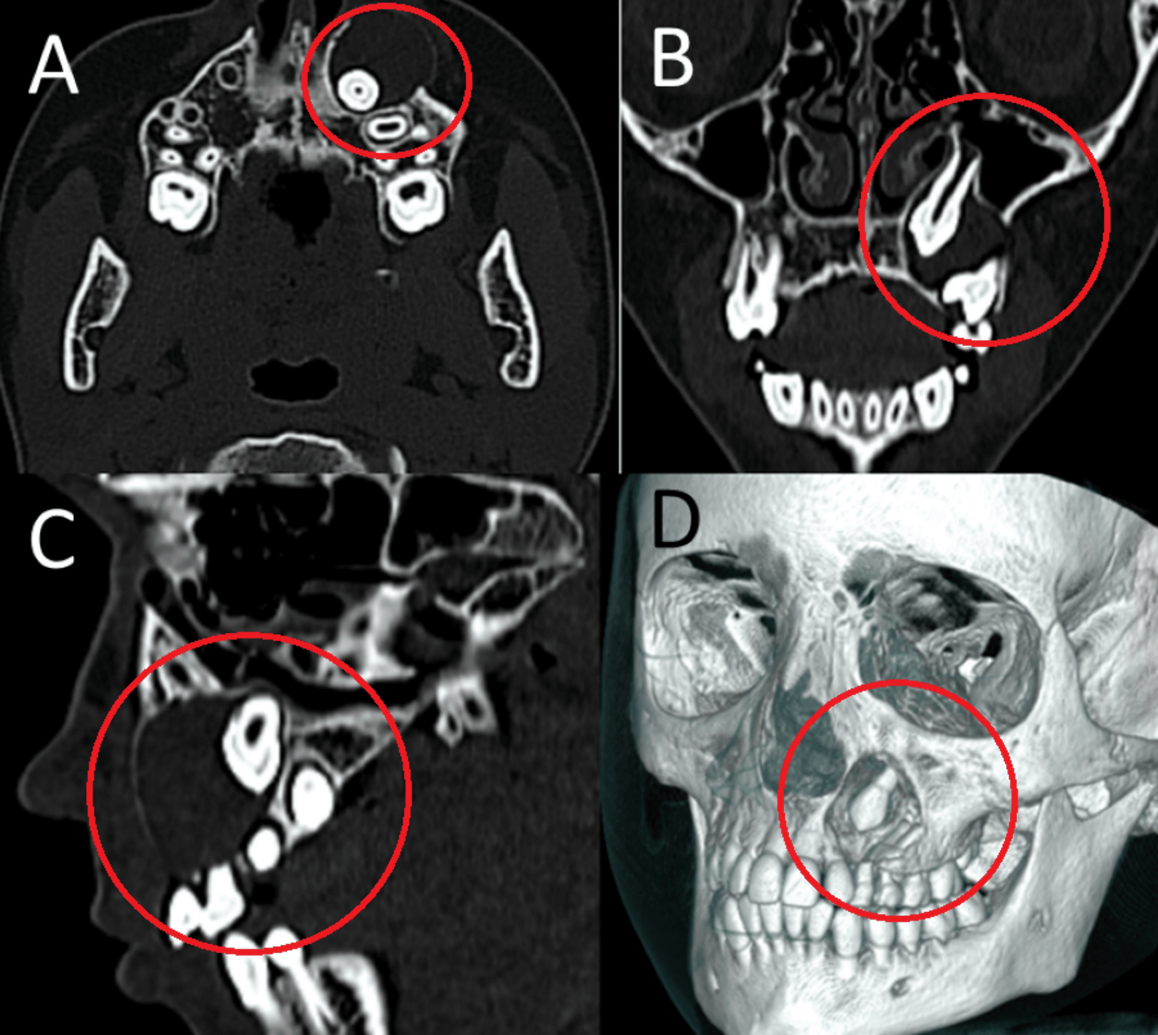
Radiographic images (A) Axial section showing root of the impacted canine inside the radiolucent cystic lesion with cortical borders (highlighted in red circle). (B) Coronal section showing cystic lesion growing into the sinus space and the lateral wall of the nose (highlighted in red circle). (C) Sagittal section showing expansion and erosion of the buccal cortical plate (highlighted in red circle). (D) Volume rendered image showing the three-dimensional location of the cyst and impacted tooth (highlighted in red circle).

Aspiration of the lesion yielded a straw-colored fluid, suggestive of a cystic lesion. Clinical examination revealed a retained left maxillary deciduous canine exhibiting Grade I mobility, while the corresponding permanent canine remained unerupted. No percussion tenderness was elicited from any of the associated teeth. CBCT imaging demonstrated a well-defined, unilocular, round-shaped radiolucency encircling the crown of the unerupted left maxillary canine, consistent with a circumcoronal cystic lesion (Figure 2).

The lesion extended from the apex of the left maxillary central incisor to the mesial aspect of the left maxillary first molar, measuring approximately 2 × 2 cm in diameter. Based on the clinical and radiographic findings, a provisional diagnosis of a dentigerous cyst was made. However, AOT was also considered in the differential diagnosis, given the patient's age, lesion location, and the presence of fine radiopaque foci within the cystic lumen, which are characteristic of AOT.

Under general anesthesia, a crevicular incision was made, and a full-thickness mucoperiosteal flap was elevated. Intraoperatively, buccal cortical plate erosion of the maxilla was observed, which provided a natural window for access to the lesion. Complete enucleation of the cyst, along with the associated unerupted canine, was performed. A peripheral ostectomy was carried out to ensure thorough removal of any residual cystic lining. The surgical site was irrigated and closed primarily using resorbable sutures. No evidence of oro-nasal or oro-antral communication was noted, and the palatal mucosa remained intact.

Histopathology findings

The excised surgical specimen was submitted for histopathological evaluation. It measured approximately 2 cm in diameter and, consistent with radiographic findings, was seen encasing the crown of the unerupted maxillary canine. The specimen was sectioned longitudinally into two halves for gross examination. Inspection revealed a cystic lesion with the unerupted canine tooth located within its lumen. The cystic lining was firmly attached at the cementoenamel junction (CEJ) of the tooth, indicative of a developmental origin. The internal (luminal) surface of the cyst appeared predominantly smooth. However, an irregular nodular area was observed on the luminal surface adjacent to the CEJ attachment site. This nodule was of particular interest and was meticulously separated from the cystic wall for detailed evaluation. Multiple representative tissue samples were taken from the luminal surface of the cyst and the isolated nodule and were submitted separately for tissue processing.

**Figure 3:**
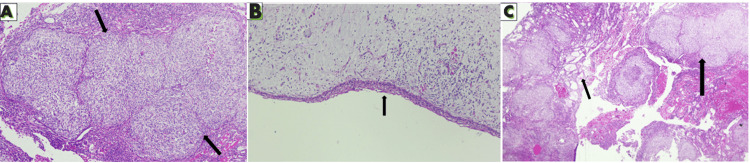
Histopathological findings (A) Epithelial nodule showing the typical whorling pattern (small arrows). (B) Cystic epithelial lining composed of thin, non-keratinized stratified squamous epithelium that is 2-4 cell layers thick and has an underlying myxomatous capsule (small arrow). (C) Neoplastic change in the cystic lesion characterized by sheets of odontogenic epithelium forming duct-like spaces (small arrow) and epithelial nodules (big arrow).

Microscopically, the lesion comprised a cystic lumen lined by thin non-keratinized stratified squamous epithelium of uniform thickness of 2-4 cell layers, with focal hyperplasia. The connective tissue capsule was myxomatous and showed subepithelial hyalinization and the presence of a few odontogenic epithelial rests (Figure 3A). Sections from the nodular area revealed sheets of odontogenic epithelium forming duct-like spaces and epithelial whorls, some with basophilic calcifications (Figure 3B). Inductive changes, including stromal hyalinization, were evident (Figure 3C). The periphery showed interconnecting strands of odontogenic epithelium. The final diagnosis was established as an AOT arising in association with a dentigerous cyst.

Postoperative follow-up

**Figure 4:**
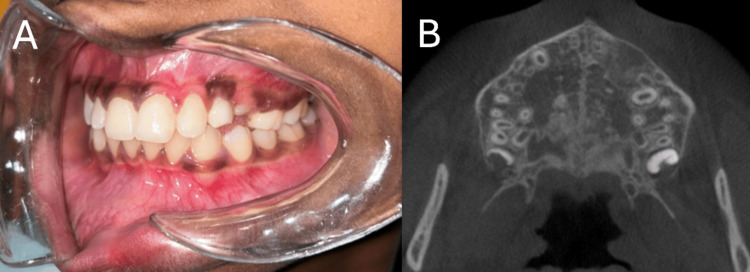
Postoperative images of six-month follow-up (A) Reduced vestibular obliteration and healed surgical site. (B) Adequate bone formation in the postoperative CBCT. CBCT: Cone beam computed tomography

The patient showed no signs of recurrence at six months postoperatively. Facial swelling and vestibular obliteration were completely gone. No pain, discharge, or paresthesia was noted. No tooth mobility was noted. Postoperative CBCT showed adequate bone formation (Figure 4).

## Discussion

According to current management protocols, enucleation of the lesion along with removal of the impacted tooth is the most widely adopted approach for treating an AOT, primarily due to its benign, encapsulated, and often cystic nature. Multiple cases have been discussed in Table 1 where the associated tooth had to be removed along with the lesion [4-14].

**Table 1:** Comparison of case reports where adenomatoid odontogenic tumors were managed conventionally and conservatively

Authors	Age	Gender	Impacted tooth	Management	Citation
Durga Sreenivas et al., 2015	14	Male	12	Enucleation + Extraction of 12,13	[4]
Kumar et al., 2015	15	Female	44	Enucleation + Extraction of 44	[5]
Uppada et al., 2015	16	Female	43	Enucleation + Extraction of 43	[6]
Majumdar et al., 2015	14	Female	23	Enucleation + Extraction of 23	[7]
Jain and Oswal, 2014	17	Female	33, 34	Enucleation + Extraction of 33, 34	[8]
Acharya et al., 2014	14	Male	13, 14, 15	Enucleation + Extraction of 13, 14, 15	[9]
Valverde et al., 2014	17	Female	23	Enucleation + Extraction of 23	[10]
Jindal et al., 2014	15	Female	43	Enucleation + Extraction of 43	[11]
Rathod et al., 2014	13	Male	34	Enucleation + Extraction of 34	[12]
Latti and Kalburge, 2013	15	Female	33	Enucleation + Extraction of 33	[13]
Kurra et al., 2013	19	Male	38	Enucleation + Extraction of 38	[14]
Taneja and Jain, 2023	13	Male	21	Enucleation + Orthodontically assisted eruption of 21	[15]
Mustakim et al., 2024	23	Female	43, 44	Curettage + Preservation of 43, 44	[16]

However, there are case reports that advocate for a more conservative approach involving the preservation of the impacted tooth and its orthodontically assisted eruption. In this case, the oblique and deep orientation of the impacted tooth made it difficult to opt for an orthodontically assisted eruption. Taneja and Jain successfully preserved and orthodontically erupted a maxillary canine associated with an AOT [15], and similarly, Motamedi et al. reported a case involving the eruption of a mandibular canine in the presence of an AOT [17]. There is another reported case by Mustakim et al. where the impacted 43, 44 were preserved following the enucleation of the lesion [16]. These cases support a conservative approach by emphasizing the hamartomatous, non-aggressive behavior of AOT rather than categorizing it strictly as a cyst or tumor.

Both AOTs and dentigerous cysts are benign, well-encapsulated lesions, for which enucleation or curettage remains the standard treatment. The prognosis following complete removal of a dentigerous cyst is excellent, with rare reports of recurrence. AOTs also demonstrate minimal aggressive potential, and recurrences are extremely rare. Typically, AOTs do not exceed 2 cm in diameter, though larger lesions, up to 6 cm, have been reported in rare cases, such as the one described by Shaikh et al. [18]. It is important to note, however, that unlike AOTs, dentigerous cysts carry a small risk of neoplastic transformation, most notably into ameloblastoma [19].

Emerging research suggests a notable association between dentigerous cysts and AOTs, with some studies proposing that AOTs may develop within the lining of pre-existing dentigerous cysts [19]. This relationship, even though rare, prompts important considerations regarding the pathogenesis of AOT and the potential, albeit low, for neoplastic transformation. It underscores the critical need for accurate diagnosis and vigilant management.

Understanding this potential overlap holds significant clinical relevance for dental professionals, oral pathologists, and maxillofacial surgeons. It highlights the importance of comprehensive histopathological evaluation to distinguish between these entities, especially in cases where AOT may mimic or coexist with a cystic lesion. Furthermore, long-term clinical and radiographic follow-up is essential to monitor for recurrence or unexpected behavior, thereby ensuring optimal patient outcomes.

Key aspects of the relationship between dentigerous cysts and AOTs include their shared origin from odontogenic epithelium, frequent association with impacted teeth, and the potential for transformation of a dentigerous cyst into an AOT. Diagnostic challenges arise due to overlapping clinical and histopathological features, making accurate differentiation critical. These factors have important implications for both treatment planning and long-term prognosis.

This complex interplay underscores the need for continued investigation to better define diagnostic criteria, optimize management protocols, and ultimately enhance patient outcomes through more tailored and evidence-based care.

## Conclusions

Analysis of the literature suggests that most clinicians prefer enucleation with removal of the impacted or affected tooth over conservative approaches such as orthodontically assisted eruption. When weighing the risk-benefit ratio, the conventional surgical method is generally favored. Although short-term outcomes following tooth retention show no immediate recurrence and a favorable prognosis for tooth vitality, the lack of long-term follow-up in existing studies limits definitive conclusions. Hence, further longitudinal research is warranted.

In the present case, the patient was reviewed first one month postoperatively and then after six months. Continued long-term monitoring is essential, given the potential for delayed recurrence in such lesions. The patient was advised to review once every six months.
